# The effect of statins on chronic obstructive pulmonary disease exacerbation and mortality: a systematic review and meta-analysis of observational research

**DOI:** 10.1038/srep16461

**Published:** 2015-11-10

**Authors:** Chao Cao, Yinfang Wu, Zhiwei Xu, Dan Lv, Chao Zhang, Tianwen Lai, Wen Li, Huahao Shen

**Affiliations:** 1 Department of Respiratory and Critical Care Medicine, Second Affiliated Hospital, Zhejiang University School of Medicine, Hangzhou, China; 2 State Key Lab for Respiratory Diseases, Guangzhou, China

## Abstract

The objective of this study is to assess whether statin use is associated with beneficial effects on COPD outcomes. We conducted a systematic review and meta-analysis of all available studies describing the association between statin use and COPD mortality, exacerbations and cardiovascular events. Medline, Embase, Web of Science, and the Cochrane Central Register of Controlled Trials were searched, with no restrictions. The hazard ratio (HR) with 95% confidence interval (CI) was estimated. Fifteen studies with a total of 238,459 patients were included. Nine articles provided data on all-cause mortality (124,543 participants), and they gave a HR of 0.62 (95% CI 0.52 to 0.73). Three studies provided data on cancer mortality (90,077 participants), HR 0.83 (0.65 to 1.08); four studies on COPD mortality (88,767 participants), HR 0.48 (0.23 to 0.99); and three studies on cardiovascular mortality (90,041 participants), HR 0.93 (0.50 to 1.72). Six articles provided data on COPD exacerbation with or without hospitalization (129,796 participants), HR 0.64 (0.55 to 0.75). Additionally, the use of statins was associated with a significant reduction risk of myocardial infarction, but not for stroke. Our systematic review showed a clear benefit of statins in patients with COPD.

Statins, 3-hydroxy-3-methyl glutaryl coenzyme A (HMG-CoA) reductase inhibitors, are commonly used in clinical practice to treat dyslipidemia^1^. In addition to the lowering of serum cholesterol, recent data indicated that statins have potent anti-inflammatory and immunomodulatory properties called “pleiotropic effects”^2^. Due to these properties, it has been suggested that these drugs may have beneficial effects in patients with chronic obstructive pulmonary disease (COPD)^3^,^4^,^5^. Most population-based observational studies have reported associations between statins and a reduced risk of mortality and hospitalization among COPD patients^6^,^7^,^8^,^9^,^10^,^11^,^12^,^13^,^14^,^15^,^16^,^17^,^18^,^19^,^20^. *In vitro* and animal studies convincingly show that statins can reduce airway inflammation by mechanisms that are unrelated to their effects on cholesterol metabolism^21^,^22^.

There is an increasing interest in determining whether statins improve the prognosis of patients with COPD. In 2009, two reviews suggested that statins might have a beneficial role in the treatment of COPD^23^,^24^. Since then, several additional trials have been conducted in this setting^7^,^8^,^9^,^11^,^12^,^17^,^19^,^20^,^25^. Overall, the results of some of the trials showed a beneficial effect, but the results were inconsistent when all trials were considered. We therefore conducted a systematic review and meta-analysis of all available studies describing the association between statin use and COPD mortality, exacerbations and cardiovascular events.

## Results

### Study selection

**Figure 1:**
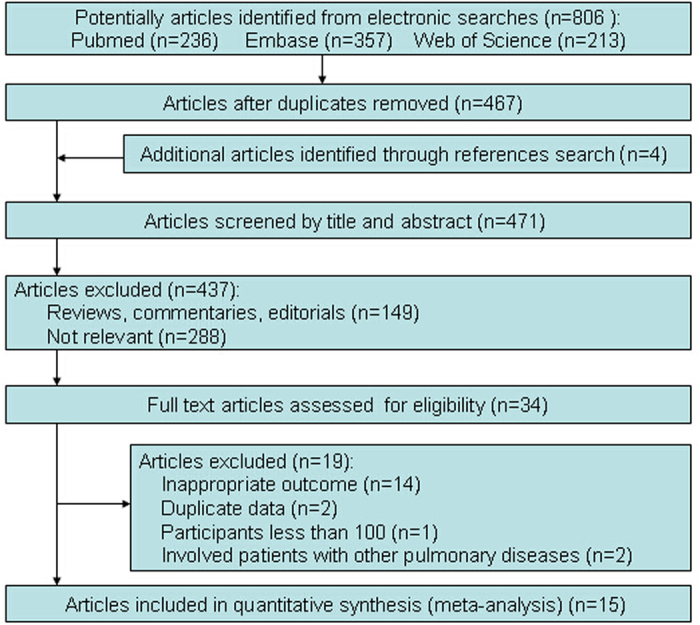
Details of literature search and study selection.

The electronic database search yielded 806 publications (Fig. 1). Another 4 studies were identified by manual searching. After exclusion of duplicates and studies that did not fulfil the inclusion criteria, 34 remaining articles were relevant for this meta-analysis. On more detailed review, an additional 19 studies were excluded for the following reasons: no outcome of interest, duplicate data, fewer than 100 participants, and the inclusion of patients with other pulmonary disease. We finally included 15 studies in our systematic review and meta-analysis.

### Study characteristics

Fifteen articles with a total of 238,459 participants with COPD were included in our study (Table 1)^7^,^8^,^9^,^10^,^11^,^12^,^13^,^14^,^15^,^16^,^17^,^18^,^19^,^20^. All patients The selected studies were published between 2006 and 2013. Most of the studies enrolled more than 1,000 participants^6^,^7^,^8^,^9^,^12^,^13^,^14^,^15^,^16^,^17^,^19^ and some of them were nationwide studies^7^,^8^,^9^,^12^,^13^,^14^,^17^,^19^. The length of follow-up and risk factors adjusted for each study are provided in Supplementary Table 1. The results of the study quality assessment showed moderate to high quality for all studies (Supplementary Tables 2, 3 and 4). Ten studies were prospective designes^6^,^7^,^8^,^9^,^11^,^12^,^15^,^16^,^19^,^20^ and five were retrospective cohorts^10^,^13^,^14^,^17^,^18^. Among these studies, one report included data from a matched cohort study and a separate case-control study, and each group was considered as a separate study in the analyses^15^. Nine studies reported all-cause mortality^6^,^7^,^8^,^9^,^10^,^11^,^12^,^13^,^14^, three reported cancer mortality^12^,^15^,^16^, four reported COPD mortality^12^,^15^, and three reported cardiovascular mortality^7^,^12^,^15^. Six cohort studies detected the effect of statin treatment on the risk of COPD exacerbation with or without hospitalization^11^,^14^,^17^,^18^,^19^,^20^. These trials were conducted in 9 countries: Canada, Norway, the Netherlands, New Zealand, Sweden, Turkey, China, the United Kingdom, and the United States.

### Effects of intervention

#### All-cause and cause-specific mortality

**Figure 2:**
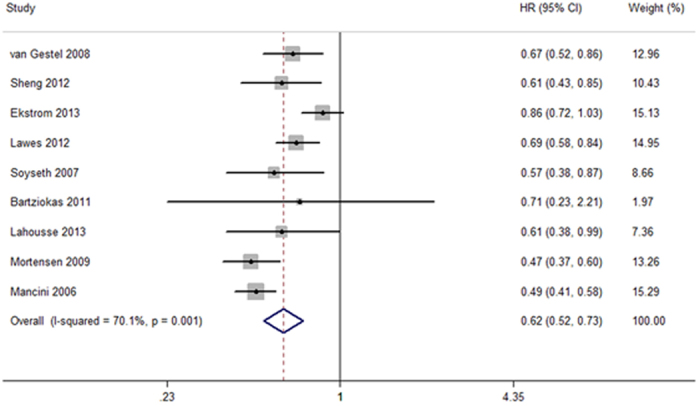
Forest plot showing effect of statins on all-cause mortality.

Table 2 shows the HRs for total and cause-specific mortality and major coronary events. The hazard ratio (HR) for all-cause mortality was significantly reduced in the statin-exposed group compared with individuals in the statin-unexposed group (HR 0.62, 95% CI 0.52 to 0.73; Fig. 2). However, no significant association of statin use with a decrease risk of cancer mortality was observed (HR 0.83, 95% CI 0.65 to 1.08; Supplementary Figure 1). In the meta-analyses of the two trials with four comparisons in COPD mortality, the HR for patients treated with statins was 0.48 (95% CI 0.23 to 0.99; Supplementary Figure 1) compared with statin non-users. For cardiovascular mortality, the HR for statin users versus statin non-users was 0.93 (95% CI 0.50 to 1.72; Supplementary Figure 1).

#### COPD exacerbation, hospitalization, and cardiovascular events

**Figure 3:**
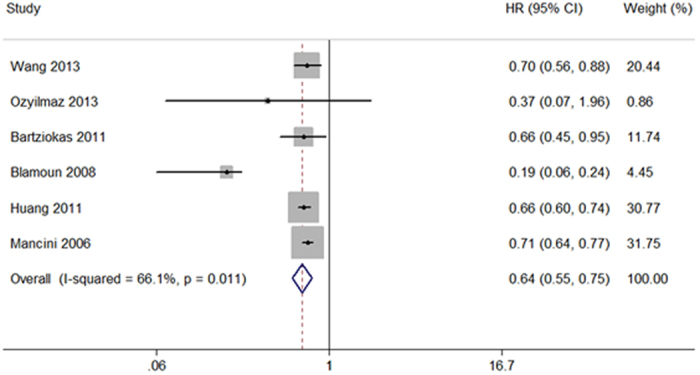
Forest plot showing effect of statins on COPD exacerbation with or without hospitalization.

The meta-analysis of six cohort studies detected a protective effect of statin treatment on the risk of COPD exacerbation with or without hospitalization (HR 0.64, 95% CI 0.55 to 0.75; Fig. 3). In the subgroup analysis, patients receiving statins presented both a lower risk of mild COPD exacerbation (HR 0.64, 95% CI 0.55 to 0.75) and a lower risk of severe COPD exacerbation requiring hospitalization (HR 0.64, 95% CI 0.55 to 0.75). Additionally, the use of statins was associated with a significantly reduced risk of myocardial infarction (HR 0.69, 95% CI 0.49 to 0.99).

### Sensitivity analysis

For all-cause mortality, the pooled result remained robust when omitting one study at a time and calculating the pooled HRs for the remainder of the studies. In a sensitivity analysis that included 7 prospective cohort studies, the pooled HR for all-cause mortality was 0.65 (95% CI 0.54 to 0.79). The study by Ekstrom *et al.*^8^ was the main origin of heterogeneity for all-cause mortality. After exclusion of this study, the heterogeneity was significantly decreased (*P* = 0.122, *I*^*2*^ = 38.6%; HR 0.58, 95% CI 0.51 to 0.66). For COPD exacerbation with or without hospitalization, the result of the meta-analysis was not affected by any single study. There was evidence of statistical heterogeneity of HR across studies (*P* = 0.011, *I*^*2*^ = 66.1%). A subgroup analysis by the severity of COPD exacerbation was performed. The heterogeneity between studies was eliminated in the severe COPD exacerbation requiring hospitalization group (*P* = 0.586, *I*^*2*^ = 0%). Three studies presented the impact of statins on all-cause mortality by relative risk (RR)^12^,^13^,^14^. After exclusion of these studies, the pooled HR for all-cause mortality was 0.71 (95% CI 0.63 to 0.81). In addition, after exclusion of moderate quality studies^10^,^13^, a statistically similar result was obtained (HR 0.65, 95% CI 0.54 to 0.79; Supplementary Figure 2).

### Analysis of publication bias

Visual inspection of the funnel plot for each outcome did not show asymmetry, suggesting no substantial evidence of publication bias. This was further confirmed by Egger's linear regression asymmetry test for each outcome (for all-cause mortality, *P* = 0.83, Supplementary Figure 3; COPD exacerbation with or without hospitalization, *P* = 0.16, Supplementary Figure 4). Funnel plots were not evaluated for secondary objectives because no other outcome had five or more available studies.

## Discussion

In this systematic review and meta-analysis of 238,459 cases of COPD from 15 articles, we found a beneficial, statistically significant association between statin treatment and COPD outcomes. Statin use was associated with a 38% reduction in all-cause mortality (95% CI 0.52 to 0.73) and a 52% reduction in COPD mortality (95% CI 0.23 to 0.99). Patients receiving statins presented a 36% lower risk of COPD exacerbation with or without hospitalization (95% CI 0.55 to 0.75).

The results observed in our review for statin use and COPD outcomes may have several alternative explanations. It is possible that there are potential beneficial effects of statins on cardiovascular comorbidities. It is known that smoking is a causative factor in the majority of patients with COPD and is a causative factor in the development of coronary artery disease. Cardiovascular comorbidities are common in COPD patients. A large cohort study involving 384,888 subjects observed that the prevalence of coronary artery disease was significantly higher among COPD patients than those in matched non-COPD controls (33.6% *vs.* 27.1%)^26^. In subjects with mild to moderate airways obstruction, every 10% reduction of forced expiratory volume in one second (FEV_1_) was associated with a 28% increase in cardiovascular mortality and a 20% increase in the risk for non-fatal coronary events^27^. The protective effect of statins could be explained by their pleiotropic effects, including anti-inflammatory, antithrombotic, and immunomodulatory effects^28^,^29^,^30^,^31^. In a randomized controlled trial, the investigators demonstrated that treatment with statins caused a significant decrease in systemic inflammation markers such as C-reactive protein (CRP) and interleukin-6 (IL-6) in patients with COPD^28^. In another report, Undas *et al.*^30^ observed that fibrin clots became more permeable, less compact, and faster lysable following three-month statin therapy in COPD patients. Additionally, it should be noted that statins could regulate the balance of Th1/Th2 cells by inhibition of Th1 development and augmentation of Th2 development of CD4 + T cells^31^. Improvements in pulmonary haemodynamics may be another potential benefit of statins in COPD^32^,^33^. Pulmonary hypertension (PH) is a common complication of COPD, and it is associated with increased risks of exacerbation and decreased survival^34^. In patients with severe COPD, statin use was associated with significantly lower pulmonary arterial wedge pressure (PAWP) and mean pulmonary arterial pressure (mPAP)^32^. Specifically, statins have been shown to inhibit the progression of PH by inhibiting endothelin-1 synthesis^33^.

The findings from our study that statins reduced mortality in patients with COPD were consistent with most of the included studies^6^,^7^,^9^,^10^,^12^,^13^,^14^. However, a large multi-centre RCT showed that statins had no effect on COPD exacerbations, which was inconsistent with our findings^25^. Several reasons might be accountable for this. Only moderate-to-severe COPD patients were included in this RCT, and it is unclear whether statins were beneficial for patients with less impairment. In addition, the mean followed-up time of this RCT was less than 2 years, which might be a significantly different compared to the long term effect. However, in this RCT, the authors did not evaluate effect of statin in the form of HR or RR, which limit our quantitative analysis with this study.

To the best of our knowledge, this is the most comprehensive systematic review and meta-analysis to date to quantitatively evaluate whether statin use is associated with beneficial effects in COPD patients. Compared with previous systematic reviews^24^,^25^,^35^,^36^, our findings extend the evidence in four ways. First, three previous studies identified half the number of studies compared with our study, and did not evaluate some clinically significant outcomes such as cause-specific mortality and cardiovascular events^24^,^25^,^35^. Second, the relationship between statins and COPD exacerbation was only investigated in our meta-analysis. Thirdly, we included only large scale studies involving a minimum of 100 participants. Eleven of the studies in our review enrolled more than 1,000 participants and eight were nationwide studies. Finally, we performed sensitivity analyses and used clinically relevant subgroups to explore the potential heterogeneity not previously reported.

The validity of a meta-analysis mostly depends on the quality of the included studies. The strengths of this meta-analysis included the large sample size of the component studies, which significantly increased the statistical power to detect potential associations. Additionally, most established risk factors for COPD were adjusted for in the fully-adjusted models in the included studies. The quality across various outcomes was moderate or high for the interested outcomes.

Several limitations of this meta-analysis are worth discussing. First, heterogeneity was observed among the included studies, although the results were generally consistent for the frequently reported outcomes. This may reflect differences in sample sizes, severity of disease, treatment strategy, and many other factors among the studies. Second, all individual studies were observational in nature but some small sample size studies (less than 100 participants) were not included in our analysis, which might lead to residual confounding or other biases. Third, three studies presented the impact of statins on all-cause mortality by RR. Considering this limitation, we further performed a sensitivity analysis, in which three studies presented with RR were removed at a time while the rest were analysed. However, statistically similar results were obtained. Fourth, optimized search strategies were required to increase the statistical power of the meta-analysis. However, to avoid missing some available publications, we used broad search strategies for studies during this step. Most studies did not report the type of statin and statin dose, which limited our further assessment of dose-response relation between statins and COPD outcomes. Nevertheless, because all studies were conducted in recent years, we believe that the patients were treated similarly based on current clinical practice recommendations. Finally, RCT is considered to be the highest-quality evidence and the gold standard for a clinical trial. However, few RCT was conducted on statins in COPD and no RCT met the inclusion criteria. Well-designed RCT of statins therapy in COPD are needed.

Our systematic review and meta-analyses showed that statin use was associated with a reduction in all-cause mortality and COPD mortality. In addition, patients receiving statins presented a lower risk of COPD exacerbation with or without hospitalization. Given the lack of evidence from multicentre randomized controlled trials, there remains a need for well-designed clinical studies on the important clinical outcomes identified, including the long term effects of statins in COPD.

## Methods

### Search strategy

We conducted the systematic review in accordance with the 2009 PRISMA (Preferred Reporting Items for Systematic Reviews and Meta-Analyses) statement (Supplementary Text 1) and the MOOSE guidelines for reporting meta-analyses of observational studies^37^,^38^. We conducted a literature search in the MEDLINE (1966 to April 2014), Cochrane Central Register of Controlled Trials (CENTRAL, The Cochrane Library Issue 1, 2014), Web of Science (1994 to April 2014), and EMBASE databases (1980 to April 2014), using the following search terms: *hydroxymethylglutaryl-CoA reductase inhibitors* or *anticholesteremic agents* or *statin* or *atorvastatin* or *cerivastatin* or *fluvastatin* or *pravastatin* or *pitavastatin* or *rosuvastatin* or *simvastatin* and *chronic obstructive pulmonary disease* or *COPD,* as well as combinations of these terms. No language restriction was applied. The abstracts of relevant scientific meetings were examined to ensure complete review of the available studies. To ensure that all relevant trials were included, we screened bibliographies of relevant review articles, and searched the clinical trial registry (www.clinicaltrials.gov) for additional studies.

### Study selection

Two investigators (CC, YW) identified suitable trials from the literature search and independently reviewed the titles and abstracts of the articles. Studies were included if they reported statin therapy in patients with COPD. We used broad inclusion criteria in this step and studies could be included by either of the two investigators. Further screening was based on full-text review. To be included, studies had to report at least one outcome of interest (mortality, acute episode of COPD, hospitalization, or cardio-cerebrovascular events) and had to enroll a minimum of 100 participants. In quantitative synthesis analysis, the outcomes that were included had at least two reports and were excluded when there was only one study. Differences between the reviewers were resolved by discussion and, if necessary, in consultation with a senior investigator (HS). In the case of multiple publications, we included the first published or more comprehensive study in the analysis.

### Data extraction and quality assessment

We abstracted information related to study design, study’s characteristics (the first author’s surname, year of publication, country, and number of participants), participants’ characteristics (age and sex), characteristics of the exposure or intervention evaluated, and information on the reported outcomes. The multivariate adjusted HR or RR and corresponding 95% CI of time-to-event data were directly extracted from the original study. When studies reported mortality at several time intervals, the longest follow-up period was selected for analysis.

Quality assessment was performed by two individual authors according to the 9-star Newcastle-Ottawa scale, which was developed to assess the quality of nonrandomized studies^39^. This scale is judged on three perspectives: the selection of the study groups (4 scores); the comparability of the groups (2 scores); and the exposure or outcome of interest (3 scores). We assigned scores of 0–3, 4–6, and 7–9 for low, moderate, and high quality of studies, respectively. Moreover, the Downs and Black scoring system was used to assess the quality of each study using the subscales involving reporting, external validity, internal validity-bias, internal validity-confounding, and power^40^.

### Statistical analysis

We used the inverse variance method where the weight of each study was proportional to the variance of the observed minus expected number of events. Patient characteristics, statin dose, and length of follow-up differed across studies, thus the multivariate adjusted outcome data were analysed to assess the HRs. For each outcome, we first assessed study heterogeneity with the Cochran Q statistic, with *P* < 0.10 indicating evidence of heterogeneity. The degree of heterogeneity was measured by the *I*^*2*^statistic, with suggested thresholds for low (25%–49%), moderate (50%–74%), and high (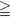
75%) values^41^. We conducted additional sensitivity analyses to explore the influence of the quality and risk of bias found in the included studies. We assessed publication bias for outcomes with funnel plots, both visually and formally with Egger’s test^42^. All analyses were performed with Stata version 12 (StataCorp, College Station, Texas). Statistical tests were judged statistically significant if the two-sided *P* value was less than 0.05.

## Additional Information

**How to cite this article**: Cao, C. *et al.* The effect of statins on chronic obstructive pulmonary disease exacerbation and mortality: a systematic review and meta-analysis of observational research. *Sci. Rep.* **5**, 16461; doi: 10.1038/srep16461 (2015).

## Supplementary Material

Supplementary Information

## Figures and Tables

**Table 1:** Characteristics of Studies Included in the Meta-analysis.

Source	Participants	Study Design	Country	Age, Mean (SD) [Range], y	Male	Statin Exposed Group	Outcome Variables
van Gestel *et al*, 2008^6^	1,310	Prospective cohort	the Netherlands	69 (9) for cases, and 69 (10) for controls	1,034 (78.9%)	Consecutive COPD patients underwent elective vascular surgery	All-cause mortality: short- (30-day) and long-term (10-year)
Sheng *et al*, 2012^7^	1,274	Population-based Prospective cohort	UK	68.5 (8.8) for cases, and 68.7 (11.8) for controls	619 (48.9%)	COPD patients in statin-exposed according to whether or not they were taking statin treatment during follow-up	All-cause mortality; Total cholesterol concentration; Cardiovascular death; Myocardial infarction; Stroke
Ekström *et al*, 2013^8^	2,249	National prospective multicenter cohort	Sweden	74.7 (8.2)	921 (41.0%)	Patients aged 45 years or older started long-term oxygen therapy for physician-diagnosed COPD	All-cause mortality
Lawes *et al*, 2012^9^	1,687	National prospective cohort	New Zealand	70.6	877 (52.0%)	Patients admitted to hospital with a first primary hospital discharge code consistent with COPD	All-cause mortality
Søyseth *et al*, 2007^10^	854	Retrospective cohort	Norway	70.8 (11.2)	414 (48.5)	Consecutive patients with a diagnosis of COPD exacerbation at discharge from hospital	All-cause mortality
Bartziokas *et al*, 2011^11^	245	Prospective cohort	Greece	71.2 (9.6)	222 (91%)	Patients admitted to respiratory medicine departments with a diagnosis of exacerbation of COPD	All-cause mortality; Adverse outcomes index (death or need for mechanical ventilation); COPD exacerbation
Lahousse *et al*, 2013^12^	2,708	Prospective population-based cohort	the Netherlands	81 [75–85] for cases 78 [74–81] for controls	1,971 (72.8%)	COPD patients had received at leastone prescription for statins between start and index date	All-cause mortality; Cardiovascular mortality; Cancer mortality；COPD mortality
Mortensen *et al*, 2009^13^	11,212	Retrospective national cohort	USA	74 (5.6)	10,993 (98.0%)	Subjects 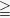 65 years of age hospitalized with a COPD exacerbation	All-cause mortality
Mancini *et al*, 2006^14^	103,004	Population-based retrospective time-matched nested case-control	Canada	77 (6) for cases，and 77 (6) for controls	43,605 (42.3%)	Patients were drawn from the the Quebec Linked Databases: two distinct COPD cohort	All-cause mortality; COPD hospitalizations; Myocardial infarction
Frost *et al*, 2007^15^	86,059	A matched cohort and two separate case-control	USA	NA	39,716 (52.1%) in cohort; 4,943 (50.3%) in case-control	COPD patients were drawn from the Lovelace Patient Database	Pneumonia and COPD mortality; Unspecified pneumonia and influenza death
van Gestel *et al*, 2009^16^	1,310	Prospective cohort	the Netherlands	69 (9) for cases, and 69 (10) for controls	1,034 (78.9%)	Consecutive COPD patients underwent elective vascular surgery	Cancer mortality
Wang *et al*, 2013^17^	7,534	Nationwide retrospective nested case-control	China	74.6 [70.0–80.6] for cases; 74.1 [69.9–80.0] for controls	6,044 (80.2%)	COPD patients were drawn from the Longitudinal Health Insurance Database	COPD exacerbation requiring hospitalization
Blamoun *et al*, 2008^18^	185	Retrospective cohort	USA	69.7 (11.6) for cases; 72.2 (10.0) for controls	119 (64.3%)	New patients admitted with a diagnosis of COPD who had been treated with statins	COPD exacerbations; Intubations secondary to COPD exacerbation
Huang *et al*, 2011^19^	18,721	Nationwide population-based prospective cohort	China	64	9,418 (50.3%)	Newly diagnosed COPD patients who received statins for hyperlipidemia treatment	Hospitalization for COPD exacerbation
Ozyilmaz *et al*, 2013^20^	107	Prospective cohort	Turkey	66.3 (8.6)	91 (85%)	Consecutive COPD patients who were admitted to out and inpatient because of COPD exacerbation	COPD exacerbation

NA = not applicable; COPD = chronic obstructive pulmonary disease.

**Table 2:** Summary of results.

Outcomes	No of pooled participants	No of trials; no of comparisons	I^2^ (%)	Pooled HR (95% CI)
All-cause mortality	124,543	9, 9^6^,^7^,^8^,^9^,^10^,^11^,^12^,^13^,^14^	70.1	0.62 (0.52, 0.73)
Cancer mortality	90,077	3, 3^12^,^15^,^16^	5.7	0.83 (0.65, 1.08)
COPD mortality	88,767	2, 4^12^,^15^	75.3	0.48 (0.23, 0.99)
Cardiovascular mortaliy	90,041	3, 3^7^,^12^,^15^	75.0	0.93 (0.50, 1.72)
COPD exacerbation	129,796	6, 6^11^,^14^,^17^,^18^,^19^,^20^	66.1	0.64 (0.55, 0.75)
COPD hospitalization	129,259	3, 3^14^,^17^,^19^	0	0.69 (0.64, 0.74)
Myocardial infarction	104,278	2, 2^7^,^14^	0	0.69 (0.49, 0.99)

NA = not applicable; COPD = chronic obstructive pulmonary disease; CI = confidence interval.
