# Supplementary material for: The effect of statins on chronic obstructive pulmonary disease exacerbation and mortality: a systematic review and meta-analysis of observational research
Source: Sci Rep. 2015 Nov 10;5:16461. doi: 10.1038/srep16461 (PMC4639730; doi:10.1038/srep16461)
Supplement: Supplementary Information [file srep16461-s1.doc]

**The effect of statins on chronic obstructive pulmonary disease exacerbation and mortality: a systematic review and meta-analysis of observational research**

Chao Cao, Yinfang Wu, Zhiwei Xu, Dan Lv, Chao Zhang, Tianwen Lai, Wen Li, Huahao Shen

| Supplementary Table 1 The length of follow-up and risk factors were adjusted for each study | |  |
| --- | --- | --- |
| **Source** | **Risk adjusted for** | **Follow-up, m** |
| van Gestel et al, 2008 | age, gender, previous myocardial infarction, previous coronary revascularization (coronary artery bypass graft or percutaneous coronary intervention), previous heart failure, previous angina pectoris, previous cerebrovascular accident or transient ischemic attack, hypertension, hypercholesterolemia, diabetes mellitus, impaired renal function, smoking status, body mass index, type of surgery, year of surgery, and use of blockers, aspirin, bronchodilators, and corticosteroids. | 60 |
| Sheng et al, 2012 | age, gender, social economic status, smoking status, home oxygen use, body mass index, FEV1 % predicted, concurrent use of drugs, comorbidity | 14 |
| Ekstro¨m et al, 2013 | age, sex, body mass index, World Health Organization performance status, resting blood gas tensions breathing air, comorbidities, and concomitant medication. | 13.2 |
| Lawes et al, 2012 | age, sex, ethnicity, history of coronary event, diabetes, prescription for β-blockers, prescription for frusemide (as a proxy for heart failure) | 48 |
| Søyseth et al, 2007 | age, gender, smoking status, concurrent use of drugs, comorbidity | 22.8 |
| Bartziokas et al, 2011 | age, gender, body mass index, current smoking status, charlson comorbidity index and COPD stage, health-related quality of life | 12 |
| Lahousse et al, 2013 | age and sex by matching; adjusted in the analyses for the use of cardiovascular drugs, antidiabetics, oral corticosteroids and duration of COPD at index date, and pack-years of cigarette smoking, total serum cholesterol, body-mass index and cardiovascular covariables at baseline. | 24 |
| Mortensen et al, 2009 | Not applicable | 3 |
| Mancini et al, 2006 | gender, age, history of prior hospitalization, congestive heart failure, or pneumonia, number of drugs used at entry, number of physician visits before entry, use of inhaled anticholinergics, steroids, and beta2-agonists, theophylline, beta-blockers, calcium-channel blockers, diuretics, nitrates, and diabetes therapies | 84 |
| Frost et al, 2007 | the number of days enrolled before and after statin initiation of the statin-exposed individual. | 12 |
| van Gestel et al, 2009 | age, gender, type of surgery, diabetes, smoking, hypercholestrolaemia, corticosteroids, statins and aspirin. | 60 |
| Wang et al, 2013 | age, stroke, hypertension, dementia, beta-blockers, vaccines, antibiotics, other lipid-lowering agents, individual types of COPD medications, and number of COPD medications | 27.6 for cases; 30 for controls. |
| Blamoun et al, 2008 | angiotensin converting enzyme inhibitors or angiotensin receptor, use of long-acting b-agonists | 12 |
| Huang et al, 2011 | age, gender, hypertension, diabetes mellitus, coronary artery disease, ischemic stroke, intracerebral hemorrhage, chronic renal disease, malignancy, heart failure, liver cirrhosis, and medication for COPD | 55 |
| Ozyilmaz et al, 2013 | Not applicable | 12 |

| Supplementary Table 2 Quality assessment of chort studies by Newcastle-Ottawa scale | | | | |  | |  |  |  |  |  |
| --- | --- | --- | --- | --- | --- | --- | --- | --- | --- | --- | --- |
| **Reference** |  | **Selection** | |  | | **Comparability** | |  | **Outcome** |  | **Overall** |
|  | *Representative of cases* | *Selection of controls* | *Ascertainment of exposure* | *Outcomes present at start of study* | | *Comparability the design or analysis* | | *Assessment of outcome* | *Adequate follow-up time （≧ 1 year）* | *Adequacy of follow up (≧ 80%)* | **Quality** |
| van Gestel et al, 2008^6^ | – | – | ★ | ★ | | ★★ | | ★ | ★ | ★ | 7 |
| Sheng et al, 2012^7^ | ★ | ★ | ★ | ★ | | ★★ | | ★ | ★ | ★ | 9 |
| Ekstro¨m et al, 2013^8^ | – | – | ★ | ★ | | ★★ | | ★ | ★ | ★ | 7 |
| Lawes et al, 2012^9^ | ★ | – | – | ★ | | ★ | | ★ | ★ | ★ | 7 |
| Søyseth et al, 2007^10^ | ★ | – | – | – | | ★★ | | ★ | ★ | ★ | 6 |
| Bartziokas et al, 2011^11^ | ★ | – | ★ | ★ | | ★★ | | ★ | ★ | ★ | 8 |
| Lahousse et al, 2013^12^ | ★ | ★ | ★ | – | | ★ | | ★ | ★ | ★ | 7 |
| Mortensen et al, 2009^13^ | ★ | – | ★ | – | | ★ | | ★ | – | ★ | 5 |
| Frost et al, 2007^15^ | ★ | ★ | ★ | ★ | | ★ | | ★ | ? | ★ | 7 |
| van Gestel et al, 2009^16^ | – | – | ★ | ★ | | ★ | | ★ | ★ | ★ | 6 |
| Blamoun et al, 2008^18^ | ★ | ★ | ★ | – | | ★ | | – | ★ | ★ | 6 |
| Huang et al, 2011^19^ | ★ | ★ | – | ★ | | ★ | | ★ | ★ | ★ | 7 |
| Ozyilmaz et al, 2013^20^ | ★ | ★ | ★ | ★ | | ★ | | ★ | ★ | ★ | 8 |

| Supplementary Table 3 Quality assessment of case-control studies by Newcastle-Ottawa scale | | | | | |  |  | |  |  |  |  |
| --- | --- | --- | --- | --- | --- | --- | --- | --- | --- | --- | --- | --- |
| **Reference** |  | **Selection** | |  | **Comparability** | | |  | | **Outcome** |  | **Overall** |
|  | *Definition of cases* | *Representativeness of cases* | *Selection of controls* | *Definition of controls* | *Comparability the design or analysis* | | | *Ascertainment of exposure* | | *Same method for cases and controls* | *Non-Response rate* | **Quality** |
| Mancini et al, 2006^14^ | – | ★ | ★ | – | ★ | | | ★ | | ★ | ★ | 7 |
| Frost et al, 2007^15^ | ★ | ★ | ★ | ★ | ★ | | | ★ | | ★ | ★ | 8 |
| Wang et al, 2013^17^ | ★ | ★ | ★ | ★ | ★ | | | ★ | | ★ | ★ | 8 |

| Supplement table 4. Quality score sheet using Downs & Black checklist | | | |  |  |
| --- | --- | --- | --- | --- | --- |
| **Source** | **Reporting (10)** | **External Validity (3)** | **Internal validity-bias (7)** | **Internal validity-confounding (6)** | **Sufficiently powered** |
| van Gestel et al, 2008 | 8 | 3 | 5 | 4 | Yes |
| Sheng et al, 2012 | 9 | 3 | 6 | 5 | Yes |
| Ekstro¨m et al, 2013 | 8 | 3 | 4 | 3 | Yes |
| Lawes et al, 2012 | 7 | 3 | 5 | 4 | Yes |
| Søyseth et al, 2007 | 6 | 3 | 2 | 2 | No |
| Bartziokas et al, 2011 | 7 | 3 | 4 | 4 | Yes |
| Lahousse et al, 2013 | 8 | 3 | 5 | 4 | Yes |
| Mortensen et al, 2009 | 6 | 3 | 3 | 2 | No |
| Mancini et al, 2006 | 8 | 3 | 4 | 3 | Yes |
| Frost et al, 2007 | 8 | 3 | 5 | 4 | Yes |
| van Gestel et al, 2009 | 6 | 3 | 3 | 5 | No |
| Wang et al, 2013 | 8 | 3 | 4 | 4 | Yes |
| Blamoun et al, 2008 | 6 | 2 | 4 | 3 | No |
| Huang et al, 2011 | 8 | 3 | 4 | 4 | Yes |
| Ozyilmaz et al, 2013 | 9 | 3 | 6 | 5 | Yes |

**Supplementary Figure legends**

**Supplementary Figure 1** Forest plot showing effect of statins on cause-specific mortality.

**Supplementary Figure 2** Sensitivity analysis of statins on all-cause mortality.

**Supplementary Figure 3** Funnel plot for studies of effects of statins on all-cause mortality.

**Supplementary Figure 4** Funnel plot for studies of effects of statins on COPD exacerbation with or without hospitalization.

**Supplementary Figure 1** Forest plot showing effect of statins on cause special mortality.


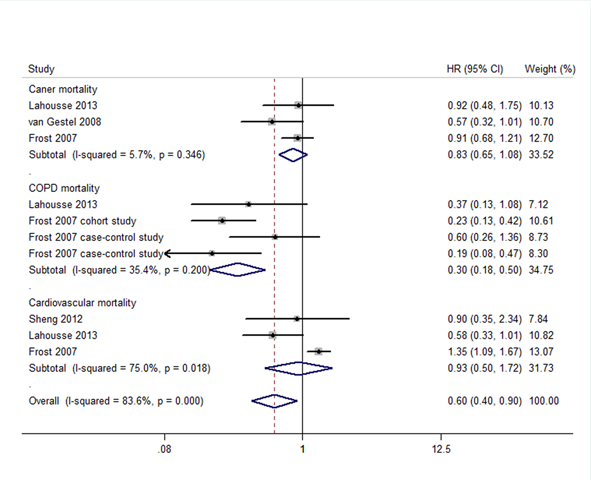


**Supplementary Figure 2** Sensitivity analysis of statins on all-cause mortality.


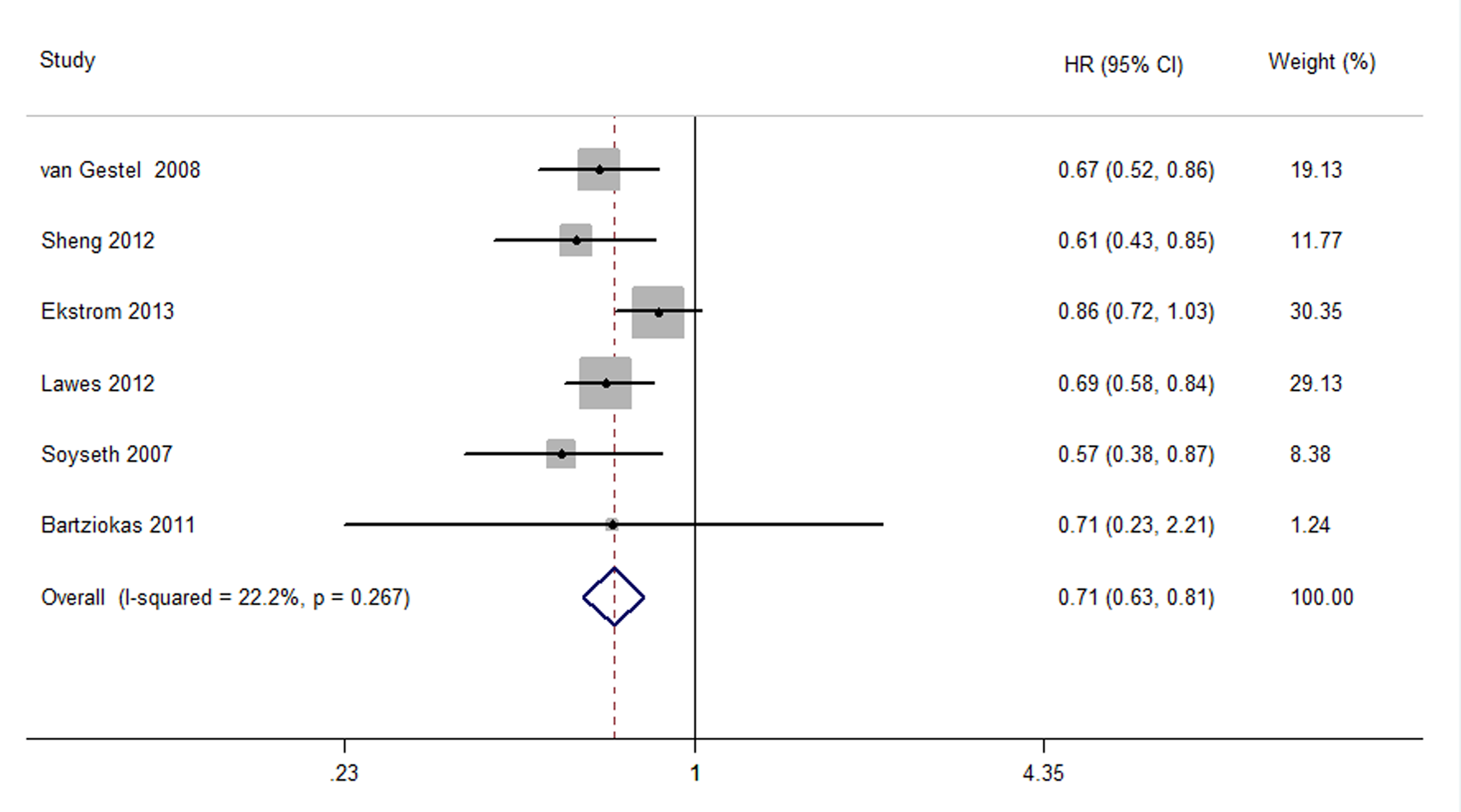


**Supplementary Figure 3** Funnel plot for studies of effects of statins on all-cause mortality.


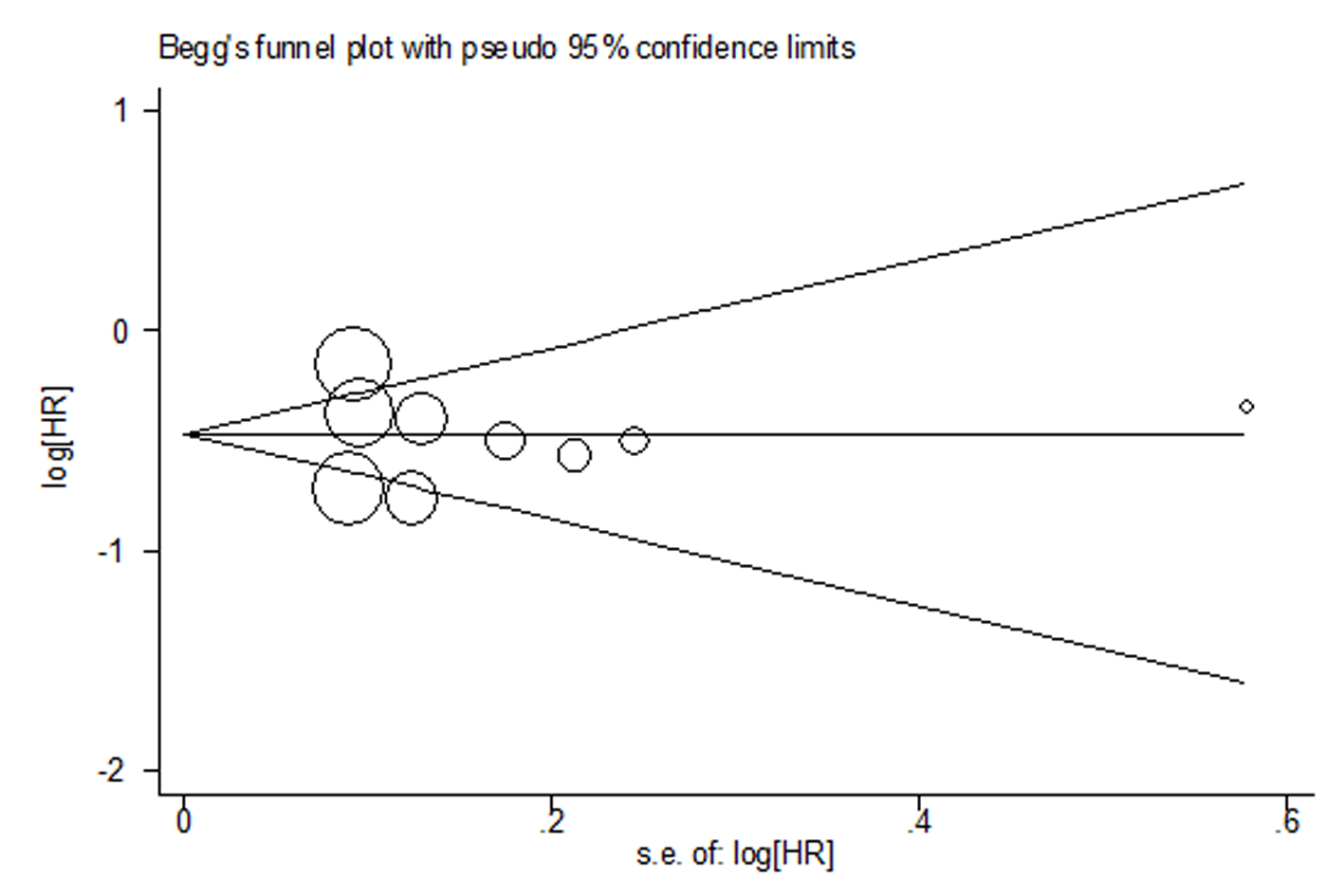


**Supplementary Figure 4** Funnel plot for studies of effects of statins on COPD exacerbation with or without hospitalization.


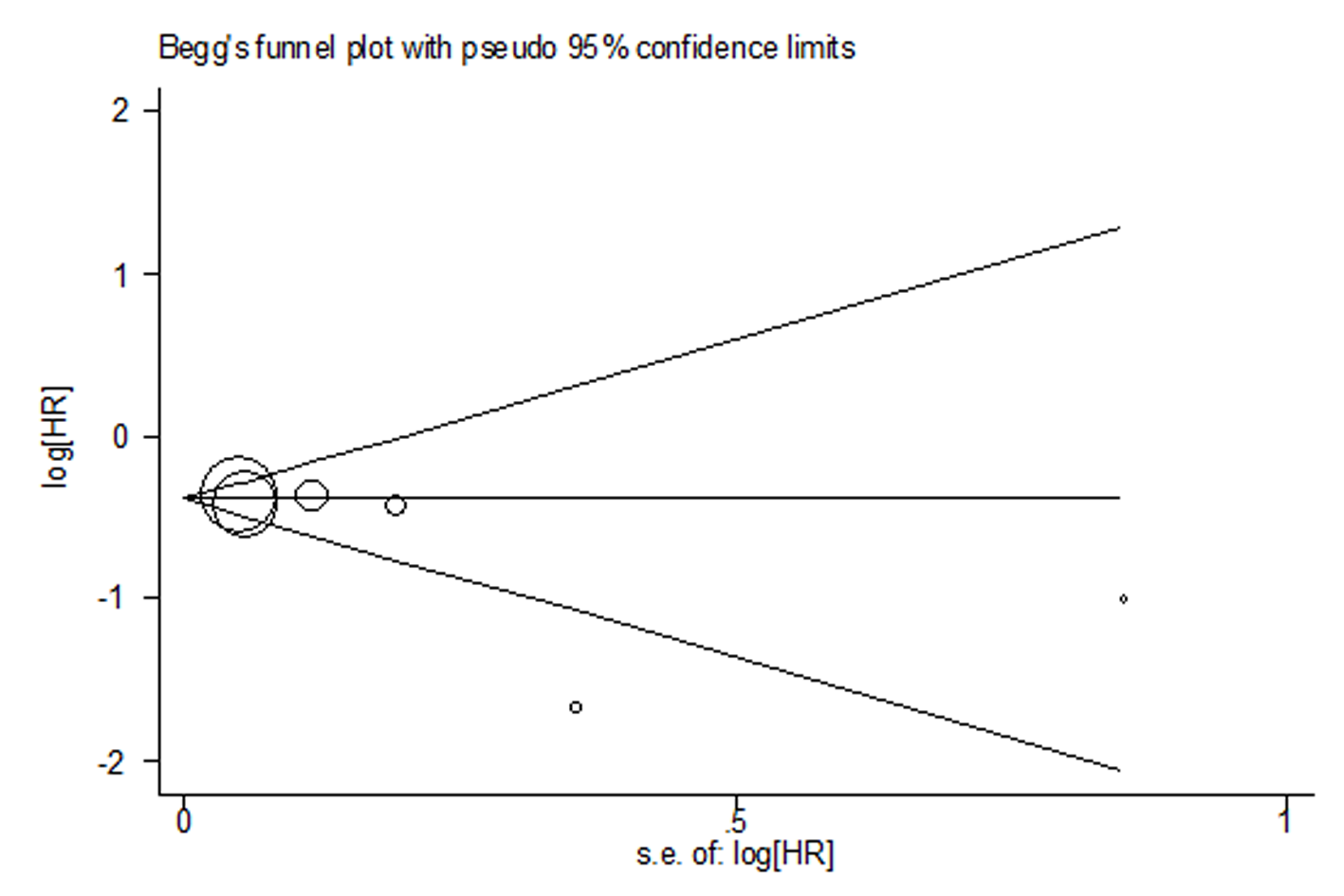


Supplementary Text 1 (PRISMA Checklist)
